# Normobaric hyperoxia-based neuroprotective therapies in ischemic stroke

**DOI:** 10.1186/2045-9912-3-2

**Published:** 2013-01-09

**Authors:** Zhifeng Qi, Wenlan Liu, Yumin Luo, Xunming Ji, Ke Jian Liu

**Affiliations:** 1 Cerebrovascular Diseases Research Institute, Xuanwu Hospital of Capital Medical University, No.45 Changchun Street, Beijing, 100053, China; 2 Central Laboratory of Shenzhen 2nd People’s Hospital, the 1st Affiliated Hospital of Shenzhen University, 3002 Sungang West Rd, Shenzhen, Guangdong, 518035, China; 3 Department of Pharmaceutical Sciences, University of New Mexico Health Sciences Center, Albuquerque, NM, 87131, USA

**Keywords:** Oxygen, Ischemia, Oxidative stress, Blood brain barrier, Reperfusion, Blood flow, Neuroprotection

## Abstract

Stroke is a leading cause of death and disability due to disturbance of blood supply to the brain. As brain is highly sensitive to hypoxia, insufficient oxygen supply is a critical event contributing to ischemic brain injury. Normobaric hyperoxia (NBO) that aims to enhance oxygen delivery to hypoxic tissues has long been considered as a logical neuroprotective therapy for ischemic stroke. To date, many possible mechanisms have been reported to elucidate NBO’s neuroprotection, such as improving tissue oxygenation, increasing cerebral blood flow, reducing oxidative stress and protecting the blood brain barrier. As ischemic stroke triggers a battery of damaging events, combining NBO with other agents or treatments that target multiple mechanisms of injury may achieve better outcome than individual treatment alone. More importantly, time loss is brain loss in acute cerebral ischemia. NBO can be a rapid therapy to attenuate or slow down the evolution of ischemic tissues towards necrosis and therefore “buy time” for reperfusion therapies. This article summarizes the current literatures on NBO as a simple, widely accessible, and potentially cost-effective therapeutic strategy for treatment of acute ischemic stroke.

## Introduction

Stroke is a leading cause of death and disability. Acute ischemic stroke results in heterogeneous changes in cerebral blood flow (CBF) and brain metabolism in the affected region [1]. As brain is extremely sensitive to hypoxia, insufficient oxygen supply due to disturbance in the cerebral blood supply is a critical event in the pathophysiology of ischemic stroke. Therefore, oxygen therapy that aims at improving tissue oxygen supply has long been considered as a logical treatment for ischemic stroke [2]. Recent animal studies have shown that short duration of normobaric hyperoxia (NBO) treatment is highly neuroprotective if started early after stroke onset [3-5]. As time loss is brain loss in the setting of acute cerebral ischemia, NBO can be administered as an early treatment to attenuate or slow down the evolution of ischemic tissue towards necrosis, and thus “buy time” for combination therapies with other neuroprotectants to achieve better outcomes [6,7]. In this review, we will summarize the current literatures on the neuroprotective studies of NBO alone and its combination with other therapies in acute ischemic stroke.

## NBO-afforded neuroprotection in stroke

### NBO: an ideal therapy for acute ischemic stroke

Oxygen, a commonly used therapeutic agent, has several properties that make it an “ideal” neuroprotective drug [8,9]. Firstly, as a small hydrophobic molecule, oxygen can easily diffuse across the blood brain barrier (BBB) to improve oxygen supply to brain tissue [10]. Secondly, short durations of oxygen are relatively safe, well tolerated, and without dose-limiting side-effects except in acute flare patients with chronic obstructive pulmonary diseases [11]. Thirdly, NBO is widely available and commonly prescribed by medical staff in a broad range of conditions to relieve or prevent tissue hypoxia. NBO can be simply administered by paramedics or even at home, which makes it an ideal acute stroke therapy. Increasing evidence from animal stroke studies has shown that NBO can reduce ischemic brain injury and improve functional outcome [12-18]. Moreover, human studies also demonstrate that NBO is associated with a transient improvement of clinical deficits and magnetic resonance imaging (MRI) abnormalities in patients with acute ischemic stroke [19-21]. The neuroprotection of NBO are explained by many possible mechanisms, such as improving metabolism, increasing CBF, reducing oxidative stress, and protecting microvasculature.

### NBO improves tissue oxygenation and metabolism in the ischemic brain

Hypoxia is a critical event leading to neuronal death in stroke. Acute ischemic stroke results in heterogeneous changes in tissue oxygenation, with a moderately reduced blood flow in the penumbra and a severely hypoperfused core [1]. Using electron paramagnetic resonance oximetry to measure localized interstitial partial oxygen (pO_2_) in a rat model, Liu et al. reported that interstitial pO_2_ rapidly decreased to about 30% of pre-ischemic values in penumbra and 4% in the core during cerebral ischemia [22]. They further showed that 95% normobaric oxygen given during ischemia was able to maintain penumbral interstitial pO_2_ levels close to the pre-ischemic value while it caused a two-fold increase in penumbral pO_2_ level if given during reperfusion [13].

However, whether NBO improves tissue oxygenation in stroke patients is still unkown. In light of studies on patients with traumatic brain injury that NBO increases brain tissue oxygen levels [23], primate or clinical studies are needed to explore brain tissue oxygenation in stroke during NBO therapy.

Abnormal metabolism is another major cause of brain injury under ischemic/hypoxic condition. NBO has been shown to reduce tissue acidosis and ATP depletion in the border zones of focal cerebral ischemia [24]. Multivoxel magnetic resonance spectroscopic imaging from stroke patients with inhalation oxygen in a flow of 45 L/min through a face mask for 8 hours indicated that lactate (a marker for aerobic metabolism) decreased during NBO administration, suggesting that NBO improves aerobic metabolism in stroke patients [21]. As the penumbra is the region that can potentially be rescued if blood flow is restored in time [25], NBO may work as an early intervention to preserve the penumbra and expand the time window of reperfusion therapies.

Inspired by recent reports on postconditioning with intermittent occlusions of cerebral artery, Liu et al. recently showed that intermittent NBO treatment (4 short cycles of intermittent NBO/air treatment) provided similar neuroprotection at 24 hours after ischemia onset and even greater neuroprotection at 72 hours, when compared to continuous NBO [26]. Their results suggest that besides providing oxygen to the ischemic tissue, intermittent NBO must have exerted its neuroprotection through other mechanisms. It is conceivable that intermittent NBO may trigger a novel form of postconditioning, in which the observed neuroprotection may be mediated by attenuating superoxide generation and activation of the Akt pathway.

These findings suggest that, in addition to improving oxygen supply and metabolism, NBO could induce endogenous protective mechanisms to reduce ischemic brain injury.

### Changes in cerebral blood flow

It is well accepted that healthy tissue responds to hyperoxia with consistent vasoconstriction [27]. How the CBF changes in ischemic tissue in response to NBO therapy is an important question. An early study from Liu et al. showed that NBO given during cerebral ischemia increased CBF in the ischemic penumbra, which was accompanied by a reduction in CBF in the contralateral hemisphere [13]. When NBO was delivered during reperfusion, it significantly decreased penumbral CBF. The blood flow in the core region was not affected by NBO during both ischemia and reperfusion [13]. Using real-time two-dimensional multispectral reflectance imaging and laser speckle flowmetry, Shin et al. reported that NBO significantly increased the CBF in ischemic cortex and reduced the cortical infarction in comparison to room air 1 hour after middle cerebral artery occlusion (MCAO) onset [28]. A study using functional cerebral blood volume (fCBV) also showed that contralateral healthy tissue responded consistently with vasoconstriction (fCBV reduction), while tissue from ischemic core showed marginal fCBV changes that later became moderate fCBV reductions. As for penumbra tissue, it showed relative preservation of mean fCBV at early time points, later exhibited significantly decreased fCBV (vasoconstriction) like healthy tissue [16].

The study from Henninger et al. indicated that NBO acutely preserved the perfusion/diffusion mismatch without altering CBF, because CBF characteristics and CBF-derived lesion volumes did not differ between NBO-treated and untreated animals [12]. The authors explained that the observed mild elevation of PaCO_2_ may have counterbalanced the vasoconstrictive effects associated with hyperoxia, suggesting that CBF changes were unlikely to be a major mechanism of neuroprotection in the acute phase of ischemia in this model.

Taken together, change of CBF in ischemic tissue in response to NBO is complicated, and whether CBF augmentation would eventually benefit stroke animals or patients needs to be investigated further.

### Impact on oxidative stress

Reactive oxygen species (ROS) may be generated when tissue oxygen level is too high or too low [29]. Oxidative stress has been widely considered as one of important mechanisms in ischemic and/or reperfusion injury [30,31]. Evidence from recent animal studies has demonstrated that short duration of NBO treatment does not increase oxidative stress if started early after stroke onset. Agardh et al. indicated that recirculation following brief periods of ischemia (15 min) did not lead to an enhanced H_2_O_2_ production with the aminotriazole/catalase method, and that hyperoxia did not aggravate the ischemic damage [32]. Liu et al. have shown that NBO given during cerebral ischemia reduces superoxide production and the generation of 8-OHdG, a biomarker of oxidative DNA damage [13]. In an attempt to identify the underlying mechanism, Liu et al. found that NBO inhibited the upregulation of gp91^phox^, the catalytic subunit of NADPH oxidase [33]. In addition, when gp91^phox^ was knocked out in the transgenic mouse model, NBO was no longer able to induce reduction in BBB leakage [14]. These results are further supported by the report that NBO treatment during focal cerebral ischemia-reperfusion does not increase oxidative stress, as measured by heme oxygenase-1 induction and protein carbonyl formation [34]. Findings from these studies indicate that NBO treatment decreases ROS production in the penumbra when penumbral pO_2_ is maintained close to the pre-ischemic level.

The effect of NBO on reactive nitrogen species (RNS) generation has also been examined in an animal stroke model. Yuan et al. recently showed NBO given during cerebral ischemia delayed and attenuated early nitric oxide generation, probably through inhibiting neuronal nitric oxide synthase (nNOS) [35]. In this study, they found that ischemia caused rapid production of NO_*x*_^−^ (nitrite plus nitrate), with a peak at 10 min after stroke onset, then gradually declining to the baseline level at 60 min. NBO delayed the NO_*x*_^−^ peak to 30 min and attenuated the total amount of NO_*x*_^−^. Moreover, NBO showed similar inhibitory effect on NO_*x*_^−^ and 3-nitrotyrosine production as that of the specific nNOS inhibitor 7-nitroindazole.

Together, these studies suggest that, contrary to common belief, under appropriate conditions NBO treatment would not cause observable increase of oxidative stress, while it may actually decrease oxidative stress in the penumbra. However, the underlying mechanism is not fully understood, and will require further investigation.

The findings from the studies on oxidative stress also suggest that the brain is more tolerable to hyperoxia than hypoxia. Although NBO treatment elevates tissue pO_2_ in the contralateral brain to a level that is much higher than the physiologic value, it did not increase ROS production [13]. Furthermore, when 95% oxygen was delivered during reperfusion, penumbral pO_2_ level was twice of the preischemic level, however no significant increase in ROS generation was observed [13]. These findings may be explained by different CBF responses to NBO in healthy, penumbral and core tissues [16]. Vasoconstriction is the normal function for healthy vasculature to avoid oxygen overload and the risk of oxidative stress. As for ischemic tissue, loss of functional vasoconstriction (vasodilation) in response to hyperoxia fortunately increases CBV and oxygen supply to overcome hypoxia. Once the tissue is rescued, the functional vasoconstriction may also return to avoid excessive oxygen supply.

### Protection on blood brain barrier

Blood brain barrier (BBB) is a physical and metabolic diffusion barrier between cerebral microvessels and the surrounding tissue, which is essential for the maintenance of homeostasis and the normal function of the central nervous system [2,36]. Matrix metalloproteinases (MMPs) have been the focus of many studies of cerebral ischemia because of their substrate specificity for fibronectin, laminin, collagen type IV and tight junction proteins (TJPs), which are structural components of the BBB [37].

In stroke animal models, the activity or expression of MMP-2 and −9 is significantly increased, which is closely related to BBB disruption, edema formation and intracranial hemorrhage [38-45]. Evidence from animal stroke studies shows that NBO inhibits MMP-9 induction in the ischemic brain, leading to reduction in occludin degradation, Evan’s blue extravasation and hemispheric swelling [3,13,33]. The mechanism by which NBO suppresses MMP-9 and attenuates BBB damage appears to involve NADPH oxidase, because i) inhibition of NADPH oxidase with apocynin or knockout of gp91^phox^ resulted in much smaller magnitudes in MMP-9 induction and BBB leakage in the ischemic brain [13,14,33], and ii) NBO did not cause additional reduction in MMP-9 induction when gp91^phox^ was knocked out [14]. These results indicate that inhibition of NADPH oxidase-derived ROS production may be an important mechanism underlying NBO-afforded BBB protection. As the tightness of the BBB is crucial to ensure the ischemic brain to safely withstand the return of blood flow, early treatment with NBO may “buy time” for thrombolytic therapy by attenuating or slowing down the disruption of the BBB.

### Controversies in NBO protection

Although a large pool of evidence has demonstrated that NBO is beneficial if it is given during cerebral ischemia, there are also controversial reports. For example, Li et al. reported that they failed to see a significant decrease in the infarct size [46]. In another study, NBO therapy for a 24 hour period was shown to reduce brain swelling in MCAO rats, but had no effect on brain infarction [46]. Perhaps, NBO for extended time (e.g., 24 hour) might reduce its neuroprotective effect. In an attempt to explore the long-term effect of NBO on ischemic brain injury, Mickel et al. showed that NBO resulted in a more severe damage to myelin sheaths in gerbils that survived 28 days after stroke onset, although neurons in the deeper laminae of the cerebral cortex appeared to be better preserved in these animals [47].

Different results have also been reported in human stroke studies regarding to NBO’s neuroprotection. Singhal et al. reported that NBO therapy via facemask for 8 hours improves stroke scale scores at 24 hours in NBO-treated patients, but not at 3 months [20]. In addition, mean relative diffusion MRI lesion volumes were also significantly reduced in NBO-treated patients at 4 hours but not subsequent time points. These findings indicated that NBO’s effects might be transient. Another study by Chiu et al. showed that breathing 40% oxygen using venturi mask decreased mortality and comorbidities in patients experiencing a first-ever large MCA infarction (more than one third of the MCA territory on brain images) within 48 hours [19]. On the contrary, Padma et al. reported that NBO in a flow of 2 L/min failed to improve the clinical scores of mean National Institute of Health stroke scale (NIHSS), modified Rankin score, Barthel index at 0, 1, 7 day of admission and at 3 months follow-up in stroke patients [48]. Obviously, better-designed clinical studies with well defined patient groups (e.g., the optimum concentration of oxygen inhalation and time after stroke onset) are needed to further investigate the safety and efficacy of NBO as a stroke therapy.

## Combination treatment with NBO

The Stroke Therapy Academic Industry Roundtable (STAIR) has strongly recommended the use of multiple neuroprotective therapies for treating stroke, in which each agent that works on a different ischemic injury mechanism could be given either simultaneously or in rapid succession [49,50]. As NBO is widely available and can be promptly initiated after stroke onset, NBO may be an ideal early stroke treatment to preserve the ischemic penumbra, and then followed by treatment with other neuroprotectants to eventually salvage the ischemic penumbral tissue.

### NBO plus tPA thrombolysis

A major goal in the treatment of acute ischemic stroke is prompt arterial recanalization. Until now, thrombolysis with tissue-type plasminogen activator (tPA) within 3 or 4.5 hour after symptom onset is the only FDA-approved treatment for acute ischemic stroke. Delayed thrombolytic therapy dramatically increases the risk of intracranial hemorrhage (ICH) [51]. As a fact, the brief therapeutic window and the high incidence of ICH have profoundly constrained the clinical use of tPA in ischemic stroke patients. Therefore, combination strategies that address not only tPA-associated ICH and its narrow therapeutic window, but also limit ischemic damage [49], are urgently needed.

Although the mechanisms underlying tPA-induced ICH are not fully understood, it has been suggested that ICH occurs as a result of BBB disruption by enhancing the proteolytic activity, such as MMPs [52-56]. Several human studies have also demonstrated that stroke patients with higher pretreatment plasma level of MMP-9 or its substrate are more likely to undergo cerebral hemorrhagic complications after tPA [57,58].

Since NBO can effectively limit ischemic neuronal death, as well as reduce BBB disruption in animal experiments [14,34], it is logic to speculate that NBO could serve as a promising adjunctive therapy to reduce tPA-associated ICH and widen the thrombolysis window in stroke. The potential for combined NBO treatment with tPA thrombolysis was recognized several years ago [15,59]. Henninger et al. showed that the combination of NBO with tPA did not increase hemorrhage volume at 10 hours or occurrence of confluent petechial hemorrhages at 24 hours in a rat embolic stroke model [60]. NBO also did not increase cellular markers of superoxide generation or brain levels of MMP-9 [61]. These data provides important initial evidence to support the combination therapy with NBO and tPA in ischemic stroke.

It was reported that combined NBO and tPA significantly reduced the mortality rate (from 70% to 11% in tPA-treated rats), brain edema, hemorrhage, and MMP-9 augmentation in a filament occlusion rat model with 5-hour ischemia followed by 19-hour reperfusion, as compared with tPA alone [6]. The authors also pointed out that they could not draw a solid conclusion on the causal relationship between mortality and cerebral hemorrhage, as they did not measure hemorrhage volume for those rats that died prematurely during reperfusion, most of which were tPA-treated normoxic rats. Nevertheless, their findings indicate that NBO can increase the safety of delayed tPA treatment. However, different results have also been reported, in which Fujiwara et al. showed that NBO plus tPA did not significantly alter hemorrhagic conversion, brain swelling, or mortality at 24 hours, as compared with tPA alone in an embolic MCAO model stroke [62]. The discrepancy might be derived from the difference in stability of animal models. Apparently, further investigation is needed to ascertain the safety and efficacy of delayed tPA treatment combined with NBO before application in human stroke patients.

### NBO plus other therapies

Minocycline, a tetracycline antibiotic, possesses properties against reperfusion injury, including anti-inflammatory, anti-apoptotic, and BBB protection. Jin et al. reported that NBO therapy after ischemia onset plus minocycline intravenous injection after reperfusion onset provides greater reduction in infarct size and edema volume. NBO plus minocycline also effectively reduces brain injury due to its inhibition on MMP-2/9-mediated occludin degradation and the activation of caspases and apoptosis inducing factor [63].

Effects of combination treatment with NBO and edaravone, a potent scavenger of hydroxyl radicals (intravenously injected after ischemia), were evaluated for reducing acute brain injury in a 2-hour filament ischemic mouse model [64]. This study indicated that combination group showed smaller infarct volume, better neurological functions and less TUNEL-positive cells in the ischemic boundary zone both in cortex and subcortex at 22 hours after reperfusion than the two monotherapy groups, suggesting that combination therapy with NBO plus edaravone prevented the neuronal damage after focal cerebral ischemia and reperfusion in mice. Combination therapy with NBO plus cilostazol, a selective inhibitor of phosphodiesterase-3, was also proved to protect the mice subjected to focal cerebral ischemia by improving CBF after reperfusion, which was partially attributed to endothelial nitric-oxide synthase activity [65].

NBO has also been suggested to combine with ultrasound and tPA in acute ischemic stroke [66]. Enhanced dissolved oxygen secondary to NBO treatment increases gas nuclei formation around and inside of the clot. Under ultrasound field, these small gas nuclei form nano bubbles that fuel inertial cavitation as substrates, and therefore increase the clot fragmentation and lysis.

Since multiple mechanisms contribute to ischemic injury, it is logic and appropriate to consider the “cocktail” approach to intervene multiple mechanisms. Moreover, combined therapies are also helpful for reducing the dosage and minimizing the toxicity of individual agent.

## Conclusions

**Figure 1:**
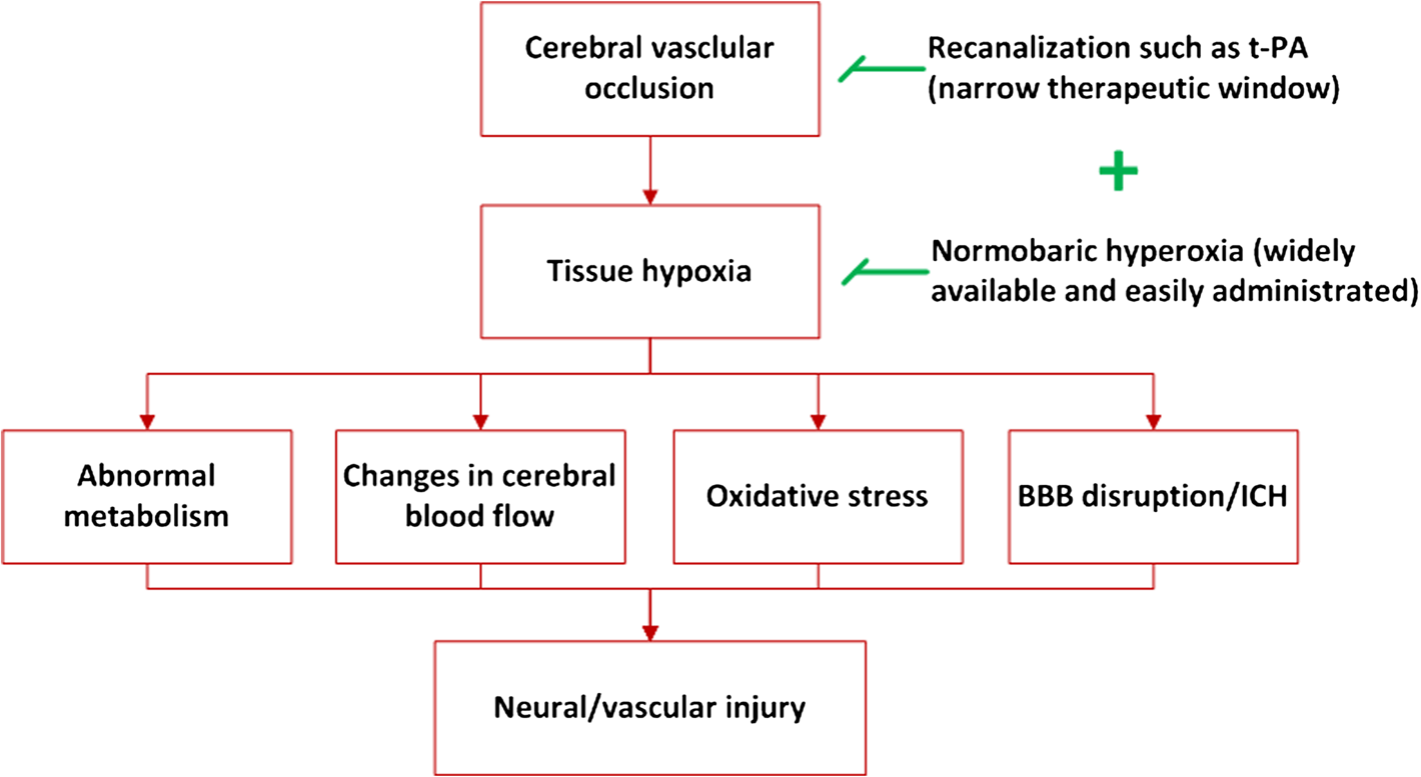
The schematic diagram of neuroprotection of NBO alone or combination therapy in ischemic stroke.

At present, stroke patients receive variable amounts of oxygen in the ambulance and current guidelines do not support the routine use of in-hospital oxygen [67,68]. In light of the positive preclinical and clinical reports, further studies are warranted to explore the efficacy of NBO therapy alone or in combination with other neuroprotectants for treatment of acute ischemic stroke, and to determine the optimum timing and duration of the therapy. NBO, a simple, widely accessible and potentially cost-effective therapeutic strategy could significantly improve the clinical outcome of stroke patients if used appropriately (Figure 1).

## Abbreviations

BBB: Blood brain barrier; CBF: Cerebral blood flow; fCBV: Functional cerebral blood volume; ICH: Intracranial hemorrhage; MCAO: Middle cerebral artery occlusion; MMPs: Matrix metalloproteinases; MRI: Magnetic Resonance Imaging; NBO: Normobaric hyperoxia; NIHSS: National Institute of Health stroke scale; nNOS: Neuronal nitric oxide synthase; NO*x*^−^: Nitrite plus nitrate; pO_2_: Partial oxygen; ROS: Reactive nitrogen species; RNS: Reactive nitrogen species; STAIR: Stroke Therapy Academic Industry Roundtable; TJPs: Tight junction proteins; tPA: Tissue-type plasminogen activator.

## Competing interests

The authors declare that they have no competing interests.

## Authors’ contributions

ZFQ reviewed the NBO studies in stroke, wrote up the manuscript and obtained partial funding. WLL edited the overall manuscript and improved the structure of this review. YML and XMJ reviewed the NBO studies in clinic. KJL participated in the overall design of this review and obtained partial funding. All authors have read and approved the final manuscript.
